# Revealing exchange bias in spin compensated systems for spintronics applications

**DOI:** 10.1038/s41598-024-76130-5

**Published:** 2024-12-28

**Authors:** Koustav Pal, Suman Dey, Aftab Alam, I. Das

**Affiliations:** 1 Saha Institute of Nuclear Physics, A CI of Homi Bhabha National Institute, Kolkata, 700064 India; 2 Department of Physics, Indian Institute of Technology Bombay, Mumbai, 400076 India

**Keywords:** Perovskite, Antiferromagnetic Exchange bias, Spin glass, Disorder, Magnetic properties and materials, Materials for devices

## Abstract

Antiferromagnetic materials offer potential for spintronic applications due to their resilience to magnetic field perturbations and lack of stray fields. Achieving exchange bias in these materials is crucial for certain applications; however, discovering such materials remains challenging due to their compensated spin structure. The quest for antiferromagnetic materials with exchange bias became a reality through our experimental study and theoretical simulation on $\hbox {Sr}_2 \hbox {FeIrO}_6$ and $\hbox {Sr}_2 \hbox {CoIrO}_6$. This study also unveils the impact of ionic disorder and lattice distortion on magnetic properties. The presence of exchange bias in both materials, given their antiferromagnetic nature, is intriguing. This study opens up new avenues for achieving exchange bias in spin-compensated systems, offering potential for low power and ultra fast antiferromagnetic spintronic applications in future research endeavors.

## Introduction

Antiferromagnetic materials show promise for future spintronic applications due to their unique attributes, including robustness against magnetic field perturbations, absence of stray fields, rapid dynamics, and capability to induce significant magnetotransport effects. Exchange bias (EB) further enhances their utility^1–4^. However, the conventional method of introducing a ferromagnetic (FM) layer in multilayer thin films to induce EB presents a drawback by adding an extra layer and increasing vulnerability to external magnetic fields^5–9^. Ideally, an antiferromagnet with EB independent of composition inhomogeneity or frustrated spins (spin glass) would be preferable. Numerous experimental and theoretical studies have illuminated the fact that the EB effect can be intentionally engineered in a plethora of heterostructures. Examples include inhomogeneous magnetic phases like FM/ferrimagnetic (FiM), FM/spin glass (SG), FM/ antiferromagnetic (AFM), hard/soft FM systems, magnetic nanoparticles, granular composites, bilayers, and superlattices^10–18^. The comprehension of these systems cannot elucidate the origin of EB in spin-compensated AFM systems which makes achieving EB in spin-compensated systems more challenging.

In a recent study involving a thin film system comprising two distinct types of AFM materials (chiral-antiferromagnet $\hbox {Mn}_3$Sn and collinear-antiferromagnet MnN), EB was observed due to the interfacial energy between the two different AFM materials^19^. However, generating EB in a single-phase AFM bulk compound presents a greater challenge. Regarding spin-compensated AFM systems, perovskite compounds play a significant role due to their remarkable ability to accommodate various ions from a wide range of the periodic table, resulting in tunable electronic and magnetic properties. With in perovskite compounds in recent times, iridium (Ir) based double perovskites, denoted as $\hbox {A}_2$*B*$\hbox {IrO}_6$ (with a specific emphasis on *B* belonging to the 3d element category), have attracted substantial attention in the condensed matter physics community. The interest of these materials arises from the fact that 3d transition metals manifest significant electronic correlation, a characteristic noticeably diminished in 5d elements. Concurrently, there is a notable increase in the strength of the spin-orbit coupling effect as the d character rises from 3d to 5d elements. There are few study on this group of 3d-5d interacting system $\hbox {Sr}_2$*B*$\hbox {IrO}_6$ (*B* = Fe, Co, Ni, Cu, Zn) which are AFM^20–23^.

In these compounds the electronic structure, as well as magnetic and transport properties, can be tuned by altering the local environment and symmetry. Through a small rearrangement of these interaction energies, intriguing physical properties such as topologically insulating behavior, superconductivity, Weyl semimetallic states, quantum spin liquid phases, etc., are predicted in these materials^24–29^. While EB is typically not expected in spin-compensated systems, few theoretical studies have proposed that EB can occur in these systems via the Dzyaloshinsky-Moriya (DM) interaction^30–32^.

The manifestation of EB via the DM interaction in bulk AFM materials has not been previously observed. Consequently, the search for new materials exhibiting EB through this mechanism is of significant interest and highly demanding. Beyond DM interaction, the profound impact of ionic disorder and structural distortion on this phenomenon is also notable. We have brought out the existence of EB in two spin-compensated Ir based compounds, $\hbox {Sr}_2\hbox {FeIrO}_6$ and $\hbox {Sr}_2\hbox {CoIrO}_6$, and examined the impact of ionic disorder and lattice distortion on their magnetic properties through experimental findings and theoretical simulations.

**Fig. 1:**
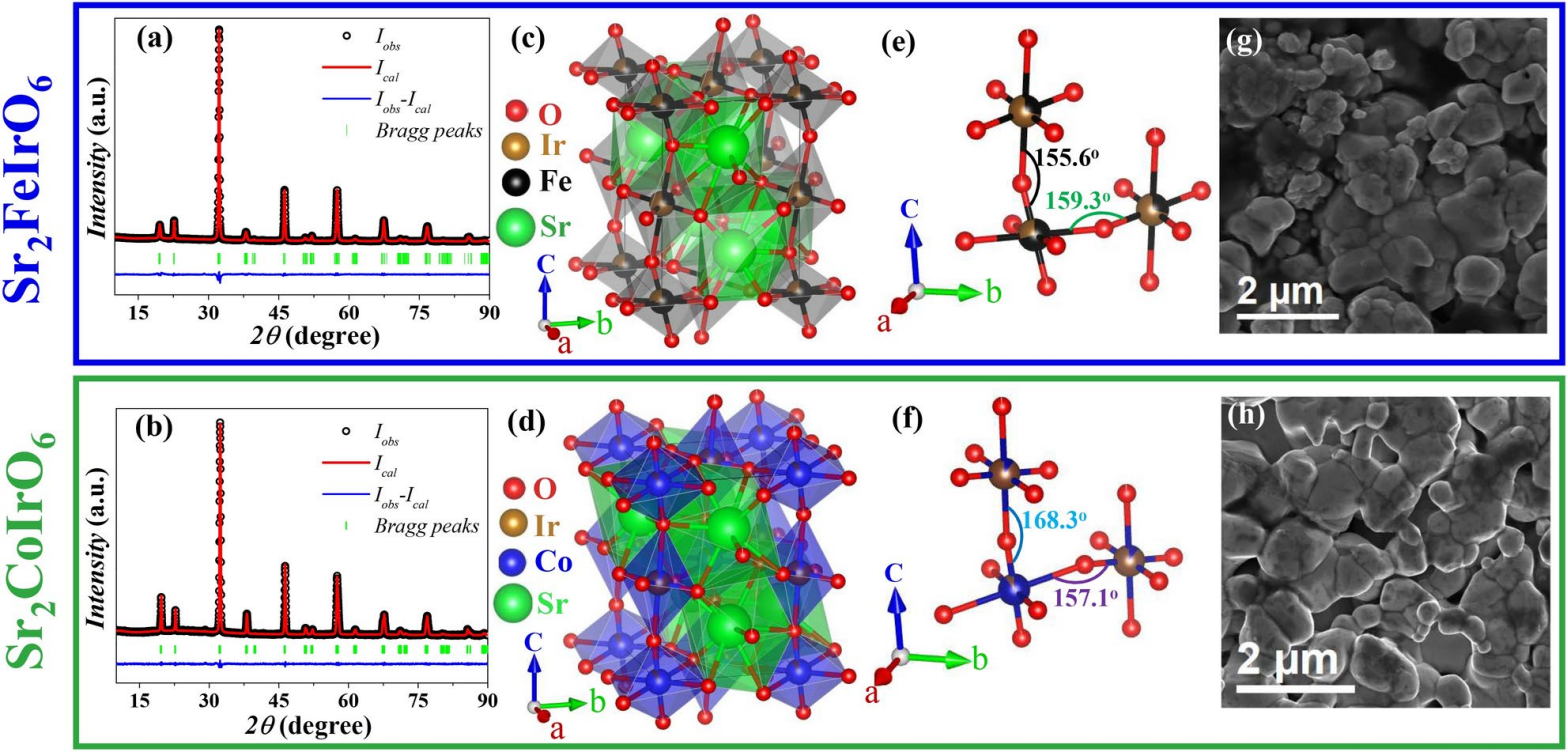
(**a**) Full Reitveld refinement of the XRD spectrum of powdered polycrystalline (**a**) $\hbox {Sr}_2\hbox {FeIrO}_6$ and (**b**) $\hbox {Sr}_2\hbox {CoIrO}_6$ compound recorded at 300 K; Triclinic crystal structure of the double perovskite (**c**) $\hbox {Sr}_2\hbox {FeIrO}_6$ and (**d**) $\hbox {Sr}_2\hbox {CoIrO}_6$ compound. The Fe(/Co)$\hbox {O}_6$ and $\hbox {IrO}_6$ octahedra are shown with Fe(/Co) (black(/blue)) and Ir (brown) at the center surrounded by the oxygen (red). Atom at the center of the cube is Sr (green); Local environment of (**e**)$\hbox {FeO}_6$, $\hbox {IrO}_6$ and (**f**) $\hbox {CoO}_6$, $\hbox {IrO}_6$; SEM Imaging of (**g**) $\hbox {Sr}_2 \hbox {FeIrO}_6$ and (**h**) $\hbox {Sr}_2\hbox {CoIrO}_6$.

## Experimental details

Polycrystalline $\hbox {Sr}_2\hbox {FeIrO}_6$ (SFI) and $\hbox {Sr}_2\hbox {CoIrO}_6$ (SCI) samples were synthesized using the solid-state reaction method. The synthesis involved meticulously mixing $\hbox {SrCO}_3$, $\hbox {Fe}_2\hbox {O}_3$, and $\hbox {IrO}_2$ powders, as well as $\hbox {SrCO}_3$, $\hbox {Co}_3\hbox {O}_4$, and $\hbox {IrO}_2$ powders, each with a purity of 99.995%, in stoichiometric proportions, followed by grinding. The mixture underwent heating at 950 °C for 16 hours, followed by regrinding and heating at 1050 °C for 24 hours. Afterward, it was regrinded, pelletized, and finally subjected to heating at 1150 °C for 72 hours. The resulting fine powder samples were analyzed using X-ray diffraction (XRD) measurements performed with a Rigaku X-Ray diffractometer, utilizing Cu-K$\alpha$ radiation. The XRD scans covered an angular range (2$\theta$) of 20–90°. Rietveld refinement technique was employed using the FULLPROF SUITE software^33,34^. The structural figures of the compounds were generated using VESTA software^35^.

The polycrystalline sample underwent extensive grinding and sonication to produce small, thin samples suitable for Transmission Electron Microscopy (TEM) imaging. Subsequently, these samples were deposited onto copper grids for TEM imaging, High-Resolution Transmission Electron Microscopy (HRTEM) imaging, Energy Dispersive X-ray Spectroscopy (EDX) analysis and elemental mapping. TEM measurements of SFI and SCI was conducted using an FEI Tecnai $\hbox {G}^2$ F30-ST instrument operating at 300 kV. This instrument was equipped with a high-angle annular dark-field (HAADF) detector from Fischione (model 3000) for Bragg scattering-free imaging and an energy-dispersive X-ray (EDX) spectroscopy attachment for compositional analysis. X-ray photoelectron spectroscopy (XPS) measurements were conducted using an Omicron Nanotechnology ultrahigh vacuum (UHV) multiprobe setup. The setup included an EA125 hemispherical energy analyzer and an Al K$\alpha$ X-ray source. The XPS data was fitted using a Shirley background and a Gaussian Lorentzian curve. The grain size of the synthesized sample was determined by analyzing scanning electron microscopy (SEM) images captured with the Zeiss Supra 40 instrument. Moreover, various magnetic measurements, including isothermal magnetization (*M*(*H*)), temperature-dependent magnetization (*M*(*T*)), and magnetic relaxation (*M*(*t*)), were conducted using a commercial SQUID magnetometer from Quantum Design.

**Fig. 2:**
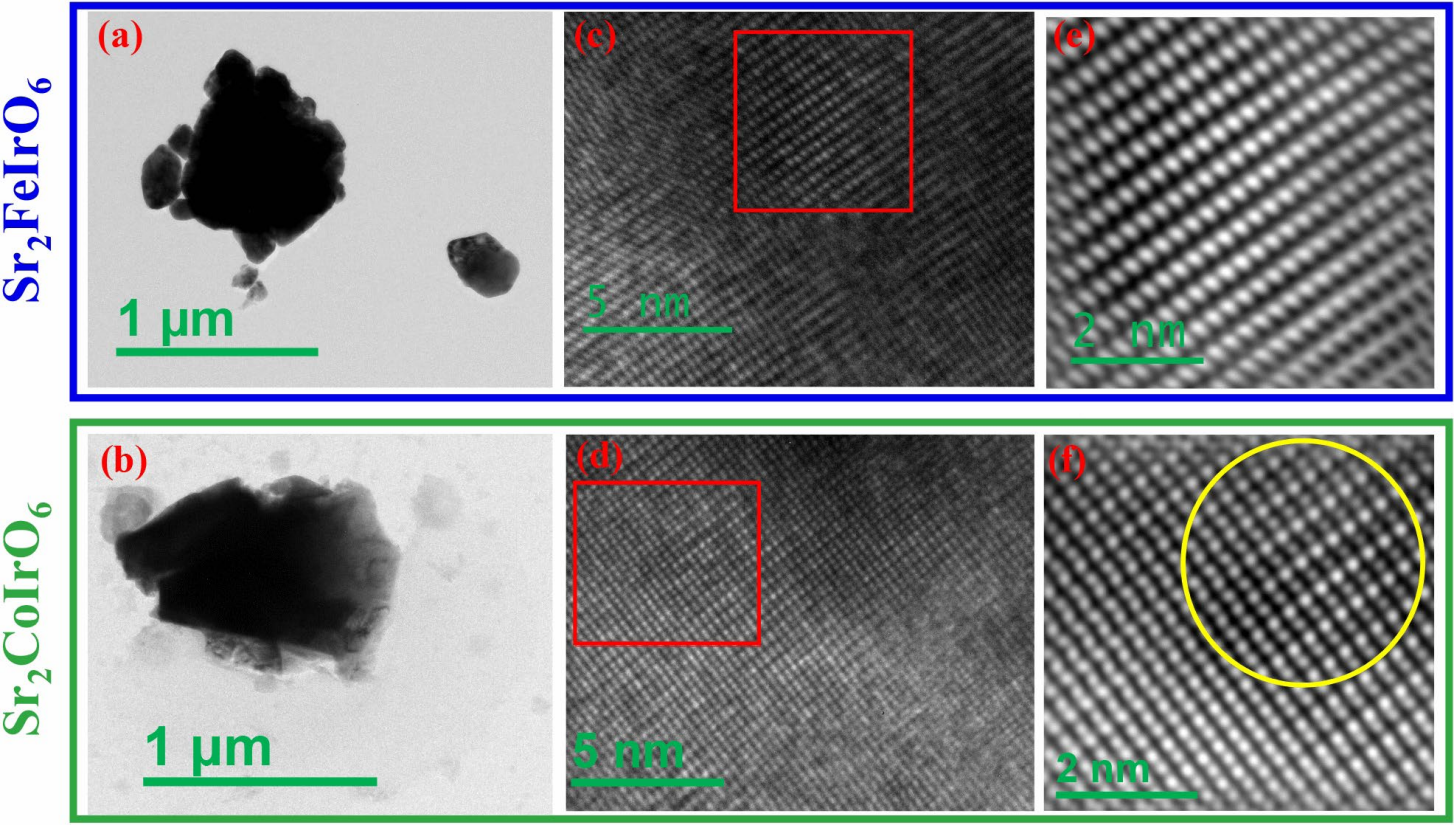
TEM image for (**a**) $\hbox {Sr}_2\hbox {FeIrO}_6$ (**b**) $\hbox {Sr}_2\hbox {CoIrO}_6$; HRTEM image for (**c**) $\hbox {Sr}_2\hbox {FeIrO}_6$ (**d**) $\hbox {Sr}_2\hbox {CoIrO}_6$; IFFT of the red box area of the HRTEM image for (**e**) $\hbox {Sr}_2\hbox {FeIrO}_6$ (**f**) $\hbox {Sr}_2\hbox {CoIrO}_6$.

**Fig. 3:**
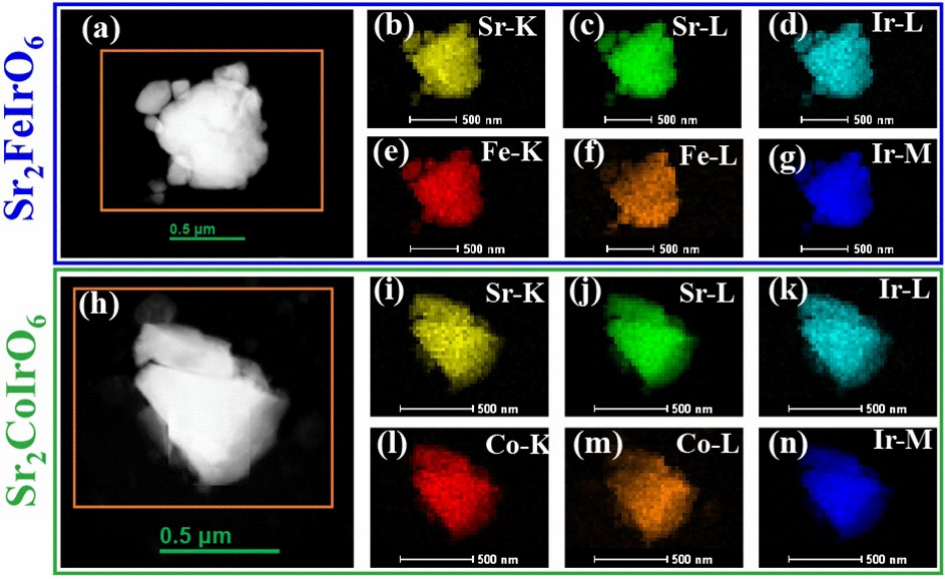
STEM-HAADF image of the particle on which Elemental mapping was performed for (**a**) $\hbox {Sr}_2\hbox {FeIrO}_6$, (**h**) $\hbox {Sr}_2\hbox {CoIrO}_6$; Elemental mapping of the constituent element for (**a**)–(**g**) Sr, Fe,Ir for $\hbox {Sr}_2\hbox {FeIrO}_6$ and (**i**)–(**n**) Sr,Co,Ir for $\hbox {Sr}_2\hbox {CoIrO}_6$.

## Results and discussions

### Structural characterization

In Fig. 1a and b, the XRD patterns of SFI and SCI are presented, accompanied by their respective full Rietveld refinement fittings. Notably, the crystal structure of the compounds crystallizes in different space groups depending on the synthesis methods and the size of the *A*-site atom^36–39^. During our analysis, we explored various possible space groups, with the best fit observed for a triclinic crystal symmetry in the I-1 space group (space group number 2)^40^. However, there remains a small peak near 28.9° that is not well-fitted with this space group, which we attribute to the influence of the silica substrate holder^41^. The lattice parameters of the structure SFI is determined to be: a = 5.55(7) Å, b = 5.55(3) Å, c = 7.86(3) Å, $\alpha$ = 89.57(1)°, $\beta$ = 90.74(3)° and $\gamma$ = 89.67(1)°. For SCI the lattice parameters are a = 5.54(4) Å, b = 5.55(2) Å, c = 7.84(1) Å , $\alpha$ = 90.02(0)°, $\beta$ = 89.67(1)° and $\gamma$ = 90.15(5)°. The refined lattice parameters, atomic coordinates and reliability factors for SFI and SCI have been presented in the supplementary Sect. I. The crystal structure includes two Wyckoff positions for Fe (Co) and Ir, specifically 2a and 2e. Thus, we performed the Full Rietveld Refinement analysis using the order parameter as a free parameter. In the SFI compound, we found a 60% ordering between Fe and Ir, whereas in the SCI compound, there is a 90% ordering between Co and Ir.

**Fig. 4:**
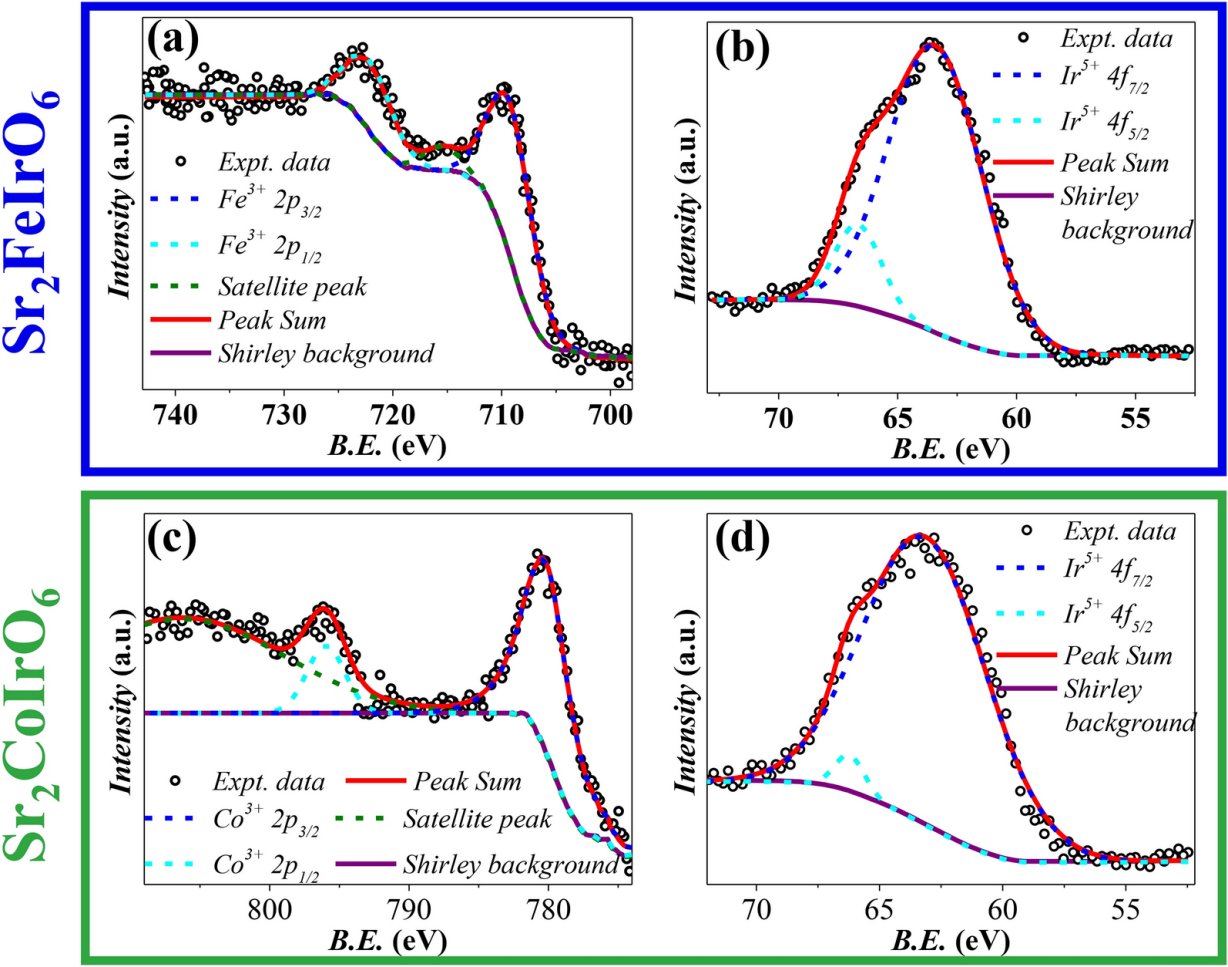
XPS spectra of core levels: (**a**) Fe 2p; (**b**) Ir 4f for $\hbox {Sr}_2 \hbox {FeIrO}_6$; and (**c**) Co 2p and (**d**) Ir 4f for $\hbox {Sr}_2 \hbox {CoIrO}_6$.

We have presented the Full Rietveld Refinement fittings at 60% and 90% order for both compounds in Supplementary Sect. I to better comprehension. Figure. 1c and d visually represent the perovskite unit cell for SFI and SCI. In the triclinic structure of SFI (SCI), the 2a(0,0,0) and 2e(0,0,0.5) sites show 60% (90%) ordering of Fe (Co) and Ir. Fe (Co) and Ir are represented by the colors black (blue) and brown, respectively, while a central Sr ion is depicted in green. From the lattice parameters, it is clear that the $\alpha , \beta$ and $\gamma$ for SCI is more close to 90° than SFI , which indicated SFI structure is more distorted than SCI. In Fig. 1e and f, the local environment of the Fe(Ir)$\hbox {O}_6$ and Co(Ir)$\hbox {O}_6$ octahedra is displayed. These octahedra exhibit distortion and disorder. In SFI structure in plane Fe/Ir–O–Fe/Ir bond angle measures 159.3° and out of plane bond angle measures 155.6°. In SCI structure in plane Co/Ir–O–Co/Ir bond angle measures 157.1° and out of plane bond angle measures 168.3°. This also indicates the higher out of plane distortion in SFI than SCI. The structural analysis clearly indicates that SFI exhibits more structural distortion and ionic disorder in the 2a–2e sites.

SEM was employed to evaluate the grain size of the synthesized sample. Figure 1g and h depict SEM images of the synthesized SFI and SCI samples respectively. The analysis reveals an average particle size in the micron range, emphasizing the bulk nature of the system.

To comprehend the crystallinity of the particles and the impact of disorder, we conducted TEM and HRTEM imaging. Figure 2 a and b depict the TEM images of the SFI and SCI compounds, respectively. The particles exhibit well-defined crystallinity up to the grain boundary, with no observable amorphous regions. HRTEM images of the SFI and SCI compounds are presented in Fig. 2c and d, respectively, revealing atomic resolution and crystalline structure. To further elucidate the details, we performed inverse fast Fourier transform (IFFT) analysis on the area marked by the red box in the HRTEM images, as shown in Fig. 2e and f. While atomic resolution is achieved for the SFI compound (Fig. 2e), the presence of substantial disorder (60% Fe and Ir) results in intensity averaging, making it challenging to distinguish individual atoms. Conversely, in Fig. 2f, distinct intensity variations are observed due to the ordered Co and Ir structure, as highlighted by the yellow circle. Based on TEM imaging, it can be inferred that SCI possesses a more ordered structure compared to SFI. We conducted elemental mapping to analyze the constituent elements of the SFI and SCI compounds. Figure 3a and h display the STEM-HAADF images of the particles on which elemental mapping was conducted for SFI and SCI, respectively. Figure 3b–g illustrate the elemental mapping of the constituent elements (Sr, Fe, and Ir) for SFI, while Fig. 3i–n depict the elemental mapping for SCI (Sr, Co, and Ir). In both instances, we observed no inhomogeneity in the images within our resolution limit. We also conducted EDAX analysis of the compound, confirming that the atomic percentage matched our chemical composition (shown in the supplementary Sect. I).

**Fig. 5:**
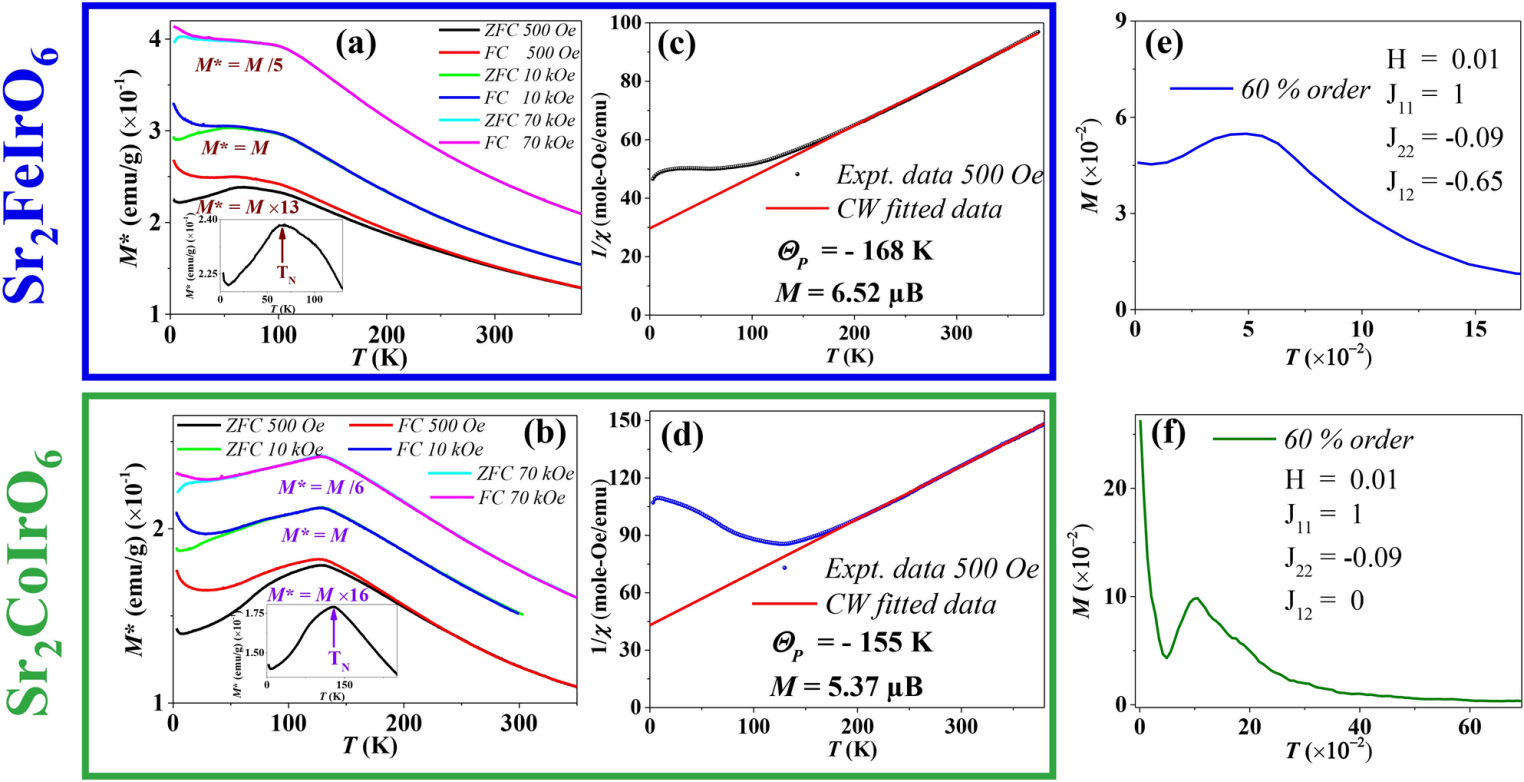
ZFC and FC *M*(*t*) of (**a**) $\hbox {Sr}_2\hbox {FeIrO}_6$ and (**b**) $\hbox {Sr}_2\hbox {CoIrO}_6$, To incorporate three *M*(*T*) plots into a single graph, we have scaled the magnetization by a factor indicated in the figure, provided just below each plot; Inverse susceptibility as a function of temperature for *H* = 500 Oe of (**c**) $\hbox {Sr}_2\hbox {FeIrO}_6$ and (**d**) $\hbox {Sr}_2\hbox {CoIrO}_6$ compound; Theoretically simulated ZFC *M*(*T*) curves with 60% disorder, where (**e**) depicts $\hbox {J}_{12}$ = − 0.65 and (**f**) depicts $\hbox {J}_{12}$ = 0.

In our exploration of the oxidation states of the metal ions within the compound, we carefully analyzed core-level XPS spectra for Fe 2p, Co 2p, and Ir 4f. Figure 4 present the analysis of Co, Fe, and Ir core-level XPS spectra for both the SFI and SCI compounds. The sample underwent X-ray irradiation, and the core-level spectra were obtained by simultaneously measuring emitted electron counts and their associated kinetic energies. These figures showcase the spin-orbit split components alongside the deconvoluted peaks.

To evaluate the ionic composition, we analyzed core-level XPS spectra for Fe 2p, Co 2p, and Ir 4f. Figure 4a and b depict the analysis of Fe and Ir core-level XPS spectra for the SFI compound. In Fig. 4a, peaks at 709.07 eV and 722.06 eV correspond to the 2*p*$_{3/2}$ and 2*p*$_{1/2}$ states of Fe, indicating $\hbox {Fe}^{3+}$ ions^42–44^. In Fig. 4b, peaks at 63.4 eV and 66.65 eV correspond to the 4*f*$_{7/2}$ and 4*f*$_{5/2}$ states of Ir, signifying $Ir^{5+}$ ions^45,46^.

Figures 4c and d illustrate the XPS analysis of Co and Ir for the SCI compound. In Fig. 4c, peaks at 780.10 eV and 795.97 eV correspond to the 2$p_{3/2}$ and 2$p_{1/2}$ states of Co, representing $Co^{3+}$ ions, with a satellite peak at 806.32 eV^47,48^. In Fig. 4d, peaks at 63.1 eV and 66.2 eV signify the 4$f_{7/2}$ and 4$f_{5/2}$ states of Ir, indicating the presence of $\hbox {Ir}^{5+}$ ions^45,46^.

###  Magnetic properties

**Fig. 6:**
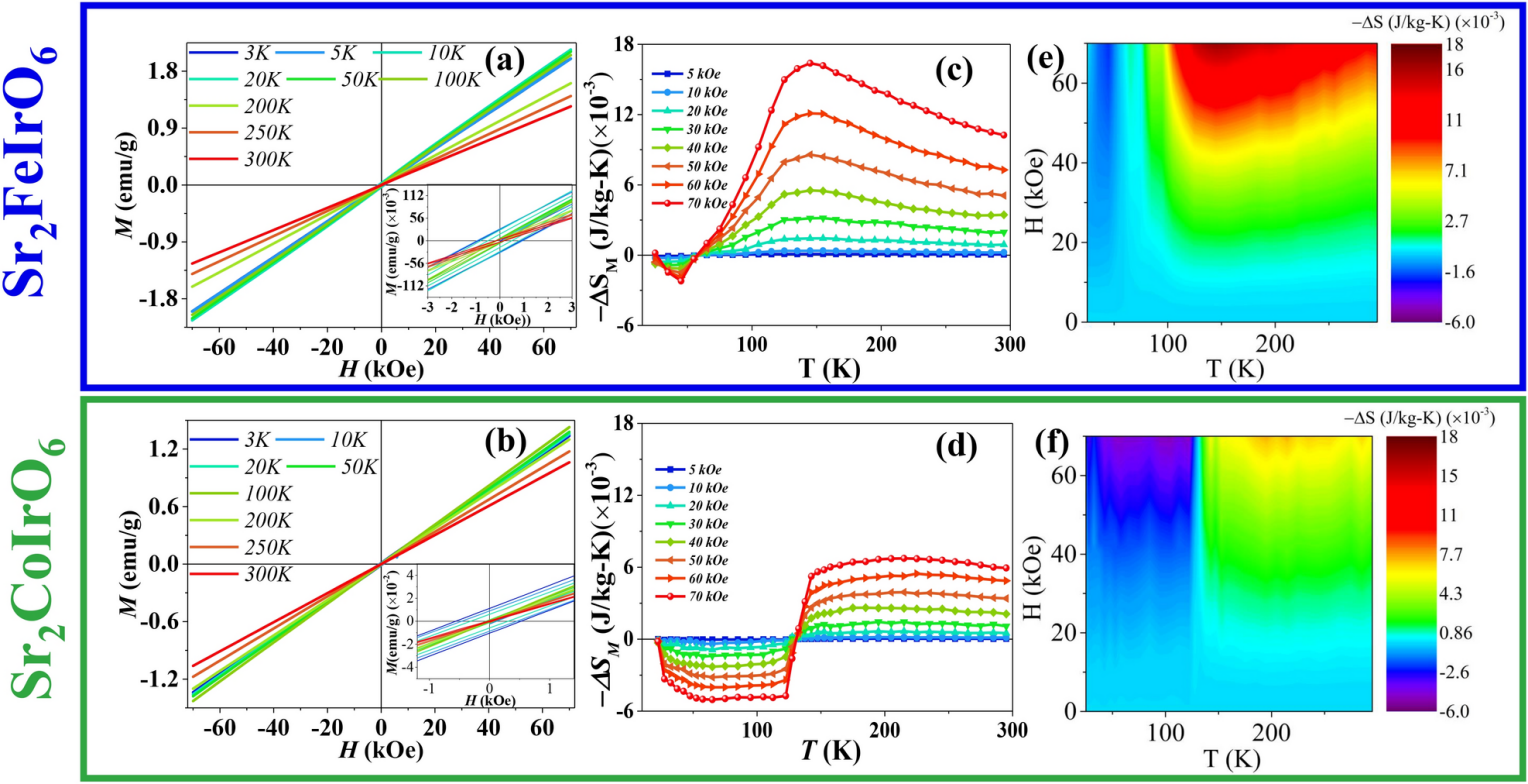
Isotherm Magnetization at different temperature for (**a**) $\hbox {Sr}_2\hbox {FeIrO}_6$ and (**b**) $\hbox {Sr}_2\hbox {CoIrO}_6$ compound. Temperature dependence of the magnetic entropy change at various applied magnetic fields for (**c**), (**e**) $\hbox {Sr}_2\hbox {FeIrO}_6$ and (**d**),(**f**) $\hbox {Sr}_2\hbox {CoIrO}_6$ compounds.

To comprehend the magnetic interactions within the system, a series of magnetic measurements were conducted. The magnetization was recorded as a function of temperature (*M(T)*), employing both the zero-field cooled (ZFC) and field-cooled (FC) protocols across a temperature range of 3-380 K at various magnetic fields. Figure 5a and b illustrate the *M(T)* plots under magnetic fields of 500 Oe, 1 Tesla, and 7 Tesla for SFI and SCI compounds. To consolidate the three *M(T)* plots into a single graph, we scaled the magnetization by a factor specified in the figure, located just below each plot ($M^{*} = M \times factor$). For SFI, the transition temperature ($T_N$) is 60 K, but for SCI, it is significantly higher at about 130 K. Although AFM interactions constitute the predominant interaction in both compounds, the somewhat smaller $T_N$ in the SFI compound raises the possibility of magnetic frustration due to its greater degree of structural disorder in the magnetic ion arrangement relative to the SCI compound. At high magnetic fields, the FC curve in the SCI compound drops around 130 K, corresponding to the AFM transition temperature. Notably, the FC *M*(*T*) curve for the SFI compound shows a trend towards saturation followed by an increase, which can be attributed to the alignment of frustrated spins along the external magnetic field direction due to the reduction in thermal energy at lower temperatures^49,50^. This behavior is characteristic of spin glass materials. In contrast, the FC *M*(*T*) curve for the SCI compound decreases after the transition temperature, driven by the alignment and anti-alignment of spins due to AFM ordering^23,51^. These results distinctly underscore the different magnetic behaviors of the two compounds. At temperatures below 15 K, both compounds display an upturn, attributable to the low crystallographic symmetry coupled with the spin-orbit coupling (SOC) present in $\hbox {Sr}_2 B \hbox {IrO}_6$ (*B* = Fe, Co) compounds. This combination facilitates a distinctive antisymmetric DM interaction, leading to the emergence of weak ferromagnetism (WFM)^52,53^. This WFM component arising from a slight canting of the antiferromagnetically ordered moments of the Fe(Co) and Ir sub-lattices at low temperatures. This behavior is reminiscent of the situation observed in Ir-based double perovskites^54^. The increase in a higher applied DC magnetic field diminishes the distinction between the FC and ZFC states at low temperature.

To enhance our comprehension of the magnetic interaction, we study the inverse susceptibility data for both compounds within the paramagnetic (PM) region and performed a fitting using the Curie-Weiss law (CW).

1 $$\begin{aligned} \chi = \frac{C}{T - \Theta _P} \end{aligned}$$

where $\Theta _P$ denotes the paramagnetic Curie temperature and *C* stands for the Curie constant. Our fitting procedure covered the paramagnetic range, extending from 175 K (190 K) to 380 K for SFI (SCI). The solid red line in Fig. 5c and d represents the closely aligned fitted data (red line) in comparison to the experimental data points (depicted by black and blue symbols for the SFI and SCI compounds). The effective PM moment ($\mu _{eff}^{exp}$) is calculated using the formula $\mu _{eff}^{exp} = \sqrt{3k_BC/N_A}$, where $k_B$ denotes the Boltzmann constant, and $N_A$ represents the Avogadro number^15^.

Based on the earlier theoretical calculations and the combined findings from the Density of States and calculated magnetic moments, we can use the result that Fe is in the +3 (3$\hbox {d}^5$) electronic state with a high spin state of S = 5/2, while Ir is in the +5 (5$\hbox {d}^4$) electronic state with a low intermediate state of S = 1^55^. Using this we can calculate the theoretical $\mu ^{\text {theory}}_{\text {eff}}$ using the formula $\mu ^{\text {theory}}_{\text {eff}} = \sqrt{(\mu _{\text {Fe}})^2 + (\mu _{\text {Ir}})^2}$, where $\mu _{\text {Fe}}$ and $\mu _{\text {Ir}}$ represent the magnetic moments of $\hbox {Fe}^{3+}$ and $\hbox {Ir}^{5+}$ cations, respectively. Applying the provided equation along with the ionic state of Fe and Ir, $\mu ^{\text {theory}}_{\text {eff}}$ is computed to be 6.56 $\mu _B$, aligning closely with the experimental value of 6.52 $\mu _B$. There are also reports suggesting that $\hbox {Ir}^{5+}$ may exist in the $S = 0$ spin state^23,56–60^. However, based on this spin state, the theoretical effective magnetic moment, $\mu ^{\text {theory}}_{\text {eff}}$, is calculated to be 5.9 $\mu _B$, which is significantly smaller than our experimental value. Consequently, we do not consider the $S = 0$ state for $\hbox {Ir}^{5+}$ in our analysis, but instead adopt the $S = 1$ state for calculations^40,55^.

For SCI, we can calculate the theoretical effective magnetic moment, $\mu _{{{\text{eff}}}}^{{{\text{theory}}}} = \sqrt {(\mu _{{{\text{Co}}}} )^{2} + (\mu _{{Ir}} )^{2} }$, where $\mu _{\text {Co}}$ and $\mu _{\text {Ir}}$ represent the magnetic moments of $\hbox {Co}^{3+}$ and $\hbox {Ir}^{5+}$ cations, respectively. Co is in the +3 (3$\hbox {d}^6$) electronic state with a ** high spin state** of S = 2, while Ir is in the +5 (5$\hbox {d}^4$) electronic state with S = 1^23,61^. Utilizing this data alongside the ionic states of Co and Ir, the theoretical effective magnetic moment ($\mu ^{\text {theory}}_{\text {eff}}$) is calculated to be 5.65 $\mu _B$, closely matching the experimental value of 5.37 $\mu _B$.

From the CW fit, we calculated $\theta _P$, for the SFI and SCI compounds as − 168 K and − 155 K, respectively. These negative values suggest that the dominating interaction is AFM. Calculating the ratio $\frac{|\theta _P|}{T_{N}}$ for the SFI and SCI compounds yields 2.8 and 1.2, respectively. This further indicates SFI compound is far more frustrated than SCI.

To investigate the relationship between $T_N$ and disorder, as well as the cause of the low temperature increment in *M*(*T*) , Monte Carlo simulations were performed. Detailed methods and the consideration of various interactions are provided in the supplementary Sect. II. To explore the effect of disorder, simulations were conducted at different percentages of magnetic ions ordering. Figure 5e presents the zero-field-cooled (ZFC) plot for $J_{11}=1.0$, $J_{22} = -0.09$, and $J_{12} = -0.65$ at $H = 0.01$, where $J_{11}$ and $J_{22}$ represent the nearest-neighbor interactions of Fe-Fe and Ir-Ir, respectively, and $J_{12}$ denotes the nearest-neighbor interaction between Fe and Ir^55^. This plot exhibits reasonable agreement with ZFC experimental data. Analyzing order percentages ranging from 100 to 60% reveals a shift of $T_N$ to lower temperatures and a decrease in the peak magnetization value with decreasing order (supplementary Sect. III). This observation supports the claim that higher disorder in SFI corresponds to a lower $T_N$. Additionally, adjusting interaction parameters elucidates their impact on $M(T)$; specifically, setting $J_{12} = 0$ amplifies the upturn at low temperatures, as depicted in Fig. 5f in contrast to our experimental observation.

To comprehend the ground state of the systems, we conducted ZFC *M(H)* measurements at different temperatures, as illustrated in Fig. 6a and b for the SFI and SCI compounds. The ZFC* M(H)* loops exhibit a symmetric nature and there is a small hysteresis in the curves, below the $\hbox {T}_{{N}}$ temperature, may indicate the presence of some WFM arising from DM interaction in AFM^62^. It is noteworthy that even under a 7-tesla magnetic field, the magnetization does not reach saturation, nor does it show any tendency to saturate. Instead, it increases linearly, indicating the presence of a strong AFM interaction.

To delve deeper into the magnetic behavior of the compounds, we conducted *M(H)* measurements at various temperatures with a temperature interval of 10 K. These measurements were used to calculate magnetocaloric effect (MCE), whose magnitude is influenced by factors like magnetic field strength, material temperature, composition etc. Examining the MCE provides valuable insights into the material’s properties, encompassing magnetic phase transitions, magnetic interactions, and entropy changes.

According to the classical Maxwell’s thermodynamic relation^63–68^, the isothermal magnetic entropy change can be expressed as follows:

$$\Delta S_{M} (T,H) = S_{M} (T,H) - S_{M} (T,0) = \int_{0}^{H} {\left( {\frac{{\partial M}}{{\partial T}}} \right)_{H} } dH{\text{ }}$$

Here, $\Delta S_M(T,H)$ represents the change in magnetic entropy, $S_M(T,H)$ and $S_M(T,0)$ are the magnetic entropies at magnetic fields *H* and 0, respectively, and $\left( \frac{\partial M}{\partial T}\right) _H$ is the partial derivative of magnetization concerning temperature at a constant magnetic field *H*. This equation establishes a connection between the change in magnetic entropy and the derivative of magnetization with respect to temperature at a constant magnetic field. A substantial change in magnetic entropy near the magnetic transition temperature can be observed due to the equation’s strong dependence on the value of $\left( \frac{\partial M}{\partial T}\right)$. The MCE is an exceptionally sensitive method for probing magnetic phase transitions in compounds. In the case of simple FM like materials, the application of a magnetic field causes the magnetic moments to become more ordered along the direction of the magnetic field. As a result, the magnetic entropy of these materials decreases in the presence of the magnetic field, leading to an increase in $-\Delta S$ and producing a peak near the FM transition temperature. Conversely, in AFM systems, the application of an external magnetic field decreases the spin fluctuation of the magnetic sublattice parallel to the direction of the field. However, the enhanced spin fluctuation of the antiparallel sublattice results in an overall increase in magnetic entropy, which causes a sudden sign reversal of $-\Delta S$ from positive to negative below the Néel temperature. This sign reversal temperature corresponds to the AFM transition temperature^63–67^.

In Fig. 6c for the SFI compound, an increase in $-\Delta S$ is observed with lowering temperature from 300K, peaking around 150 K, followed by a gradual decline. $-\Delta S$ changes sign from positive to negative around 60 K, near the $\hbox {T}_{N}$ temperature. Figure 6e illustrates a 3D plot, depicting a hump-like gradient around 150 K and a slow, steady change. The positive $\Delta S$ signifies strong AFM correlations. However, the gradual variation also indicates the presence competing interaction in the compound. In Fig. 6d, the MCE for the SCI compound is depicted. Instead of showing a distinct peak, there is a sudden jump in $-\Delta S$ from a positive to a negative value around 130 K. Figure 6f further elucidates this abrupt change around 130 K with lowering temperature, indicating the presence of strong long-range AFM ordering.

**Fig. 7:**
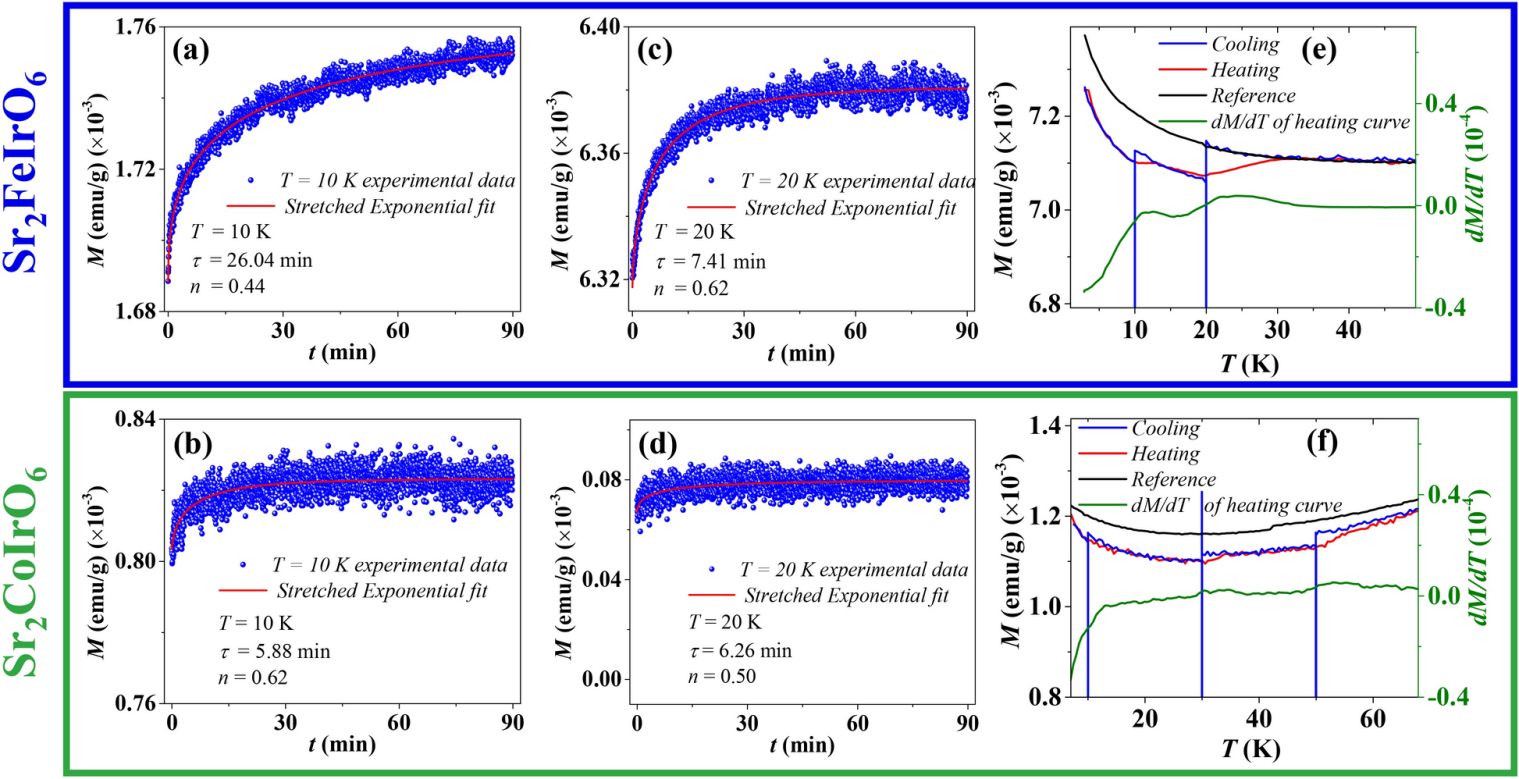
The relaxation curves for $\hbox {Sr}_2\hbox {FeIrO}_6$, obtained from the ZFC protocol at (**a**) *T* = 10 K and (**c**) *T* = 20 K in the presence of *H* = 100 Oe; The relaxation curves for $\hbox {Sr}_2\hbox {CoIrO}_6$, obtained from the ZFC protocol at (**b**) *T* = 10 K and (**d**) *T* = 20 K in the presence of *H* = 100 Oe; Experimental data were fitted (red line) using Stretched Exponential function; Memory effect of (**e**) $\hbox {Sr}_2\hbox {FeIrO}_6$ and (**f**) $\hbox {Sr}_2\hbox {CoIrO}_6$ compound, where stops administered during cooling are recovered in heating, as seen in the derivative plots. A reference curve without stops is also presented for comparison.

To identify the presence of the glassy phase, we conducted magnetic relaxation ($M(t)$) and magnetic memory experiments. Both ZFC and FC protocols are commonly employed to investigate $M(t)$ behavior. For this study, we chose to utilize the ZFC protocol. The system was cooled from its paramagnetic state (T > 350 K) to the desired temperature without the application of an external magnetic field, allowing for a brief period to ensure temperature stabilization. Subsequently, the time-dependent magnetization was measured over an extended duration while applying a small magnetic field of 100 Oe.

Figure 7a and c depict the acquired relaxation curves for the SFI compound over a time period of time 5400 s at 10 K and 20 K, respectively. These curves demonstrate non-saturating and logarithmic behavior over time at the specified temperatures. Such slow magnetization evolution arises from thermal or quantum transitions amid numerous potential energy barriers, each associated with distinct relaxation times.

In glassy states, magnetic relaxation is described by stretched exponential functions, as expressed by the equation:

$$\begin{aligned} M(t) = M_0 + M_g \exp \left( - \left( \frac{t}{\tau }\right) ^n \right) \end{aligned}$$

Here, $M(t)$ signifies the magnetization at time $t$, $M_0$ represents the initial magnetization, $M_g$ denotes a fitting parameter related to glassy component of magnetization, τ stands for the characteristic relaxation time, and $n$ indicates the stretching exponent. This equation provides a mathematical representation of the observed time-dependent decay of magnetization in magnetic relaxation experiments.

To compare the relaxation behavior, it is essential to perform measurements for both compounds at the same temperature. However, since the transition temperature of SFI is nearly half that of SCI, we cannot conduct these experiments at higher temperatures. Additionally, we aim to perform the measurements at temperatures that allow sufficient intervals between consecutive data points. Therefore, we carried out the experiments at 10 K and 20 K, both well below the transition temperatures of both compounds. Furthermore, since 20 K is twice the value of 10 K, it provides a clear understanding of the temperature dependence.

For the SFI system at 10 K, τ is remarkably long, with a value of 26 minutes. The calculated *n* value is approximately 0.44, but with an increase in temperature to 20 K, τ is only 7 min., which is almost quadruple times less than the previous value. The observed magnetic relaxation behavior presents compelling evidence supporting the existence of a glassy phase in the SFI system at low temperature.

In contrast, SCI does not possess such a long relaxation time at 10 K and 20 K shown in Fig. 7b and d. The τ at 10 K is only 2.8 min with *n* value of 0.43. It does not change much at high temperatures. At 20 K, τ is 2.1 min, and $\beta$ is 0.3. It clearly shows that SCI does not possess a glassy nature like SFI.

**Fig. 8:**
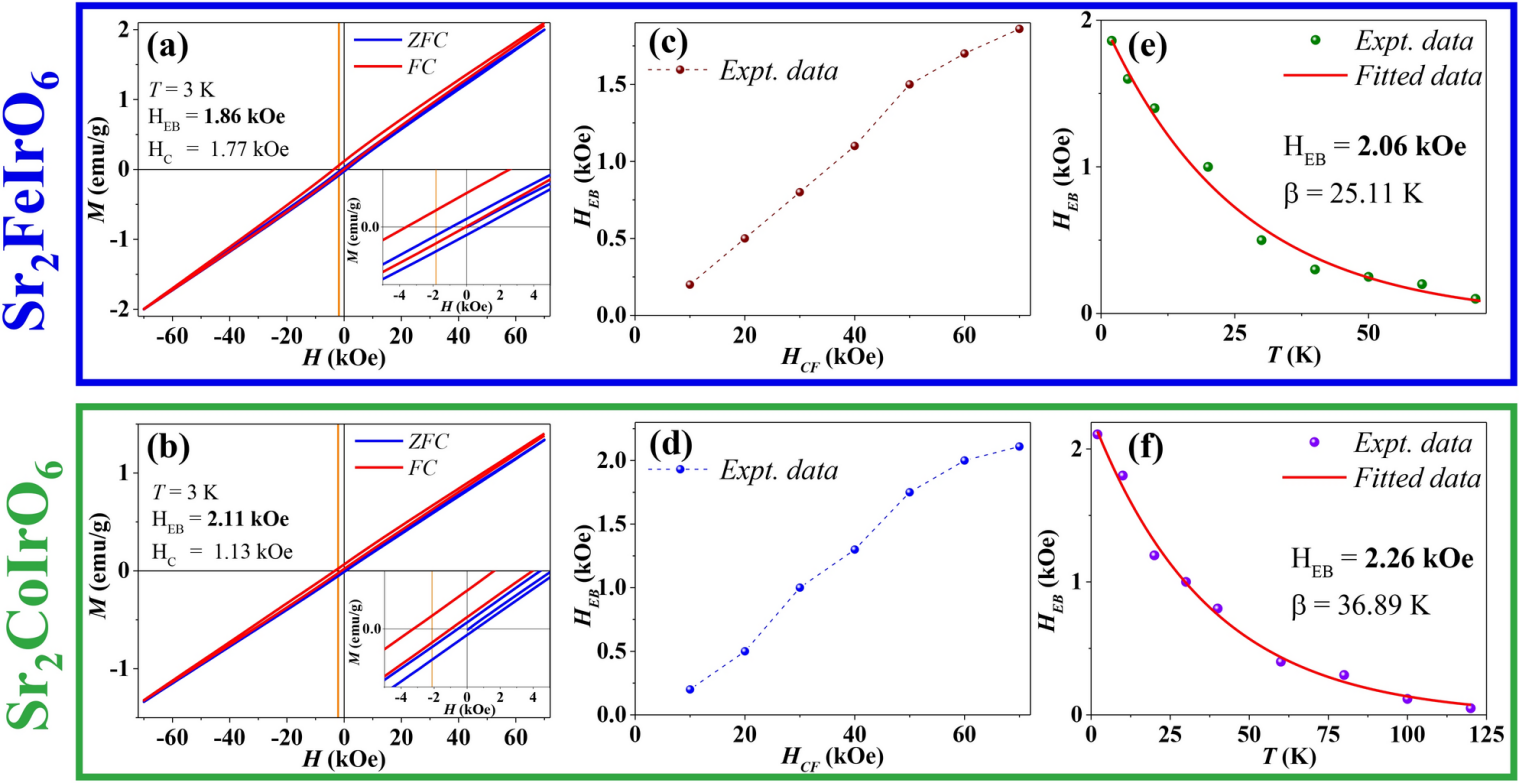
*M*(*H*) at *T* = 3 K in both ZFC and FC modes for (**a**) the $\hbox {Sr}_2\hbox {FeIrO}_6$ and (**b**) the $\hbox {Sr}_2\hbox {CoIrO}_6$ compound; and the dependence of EB on $\hbox {H}_{{FC}}$ and temperature for (**c**) and (**e**) for the $\hbox {Sr}_2\hbox {FeIrO}_6$ compound, and (**d**) and (**f**) for the $\hbox {Sr}_2\hbox {CoIrO}_6$ compound.

To understand the difference in relaxation behavior between these two compounds, we conducted magnetic memory experiments, a captivating phenomenon observed in frustrated spin systems. In the memory experiment, the sample is first cooled down to a low temperature at a constant rate while measuring the magnetization. During this cooling process, intermediate stops are introduced at regular intervals (45 min) below $T_{\text {N}}$, allowing the magnetization to relax. Once the temperature of 5 K is achieved, the sample is heated back up without any stops, and the magnetization is recorded. For comparison purposes, a reference curve is recorded with no intermediate stops. The cooling, heating, reference, and the derivative of the heating curve ($\frac{dM}{dT}$) are presented in Fig. 7e and f for the SFI and SCI compounds.

For SFI compound the cooling curve shows distinct “dips” in magnetization where the measurement was halted, while the heating cycle reveals signatures of the memory effect where the steps are retrieved at the same temperature points. These are clearly visible in the $\frac{dM}{dT}$ plot. On the contrary, the memory effect in the SCI compound is not distinctly visible, and in the $\frac{dM}{dT}$ plot, it appears nearly flat. The relaxation and memory effect experiments clearly indicate that SFI possesses a more pronounced glassy nature than SCI.

Based on the conducted experiments and analyses, it has been observed that there is a pronounced presence of strong AFM interaction in both compounds. However, what’s particularly intriguing is that SFI exhibits disorder and spin glass behavior, whereas SCI does not manifest such behavior. In this context, it is interesting to investigate the presence and origin of EB in both systems. The shift of a material’s hysteresis loop along both the field and magnetization axes can be conventionally described using two parameters. The first parameter is the EB field ($|H_{EB}| = |H_+ + H_-|/2$), quantifying the magnitude of the shift. The second parameter is the coercive field ($|H_C| = |H_+ - H_-|/2$), representing the strength of the magnetic field required to demagnetize the material. The first and second intercepts of the magnetization curve with the field axis are denoted by $H_+$ and $H_-$, respectively.

ZFC and FC $M(H)$ measurements were conducted at 3 K using both ZFC and FC protocols. Figure 8a and b illustrate the $M(H)$ curves for the SFI and SCI compounds at 3 K with a magnetic field strength of 7 Tesla. The magnitudes of $|H_{EB}|$ and $|H_C|$ were determined from the $M(H)$ loops to be approximately 1.86 kOe and 1.77 kOe, respectively, for SFI. For SCI, the magnitudes of $|H_{EB}|$ and $|H_C|$ were determined to be 2.11 kOe and 1.13 kOe, respectively. The measurement of FC $M(H)$ has been conducted during the cooling process under both +7 Tesla and -7 Tesla magnetic fields. In each case, the value of the EB has yielded nearly identical values.

To investigate the temperature and cooling field ($\hbox {H}_{{CF}}$) dependence on EB, measurements were performed at 3 K with varying $\hbox {H}_{{CF}}$ and at different temperatures with a constant $\hbox {H}_{{CF}}$. Figure 8c and d illustrate the $\hbox {H}_{{CF}}$ dependency at 3 K. In both cases, EB increases with increasing $\hbox {H}_{FC}$.

Figure 8e and f depict the temperature dependence of $H_{EB}$ for SFI and SCI compound, respectively. As temperature increases, $H_{EB}$ exhibit a decrease and their behavior is effectively described by the following equations:

2 $$\begin{aligned} H_{\text {EB}}(T) = H_{\text {EB}}(0) \exp (-T/\beta ) \end{aligned}$$

Here, $\beta$ represents a constant, and $H_{\text {EB}}(0)$ denotes the extrapolation of $H_{\text {EB}}$ to absolute zero temperature. The values of $H_{\text {EB}}$ and $\beta$ for SFI are 2.06 kOe and 25.11 K, respectively. For SCI, the values of $H_{\text {EB}}$ and $\beta$ are 2.26 kOe and 36.89 K, respectively. The variation of the EB field with temperature suggests in case of SCI with increasing temperature EB decreases slowly compared to SFI.

### Discussion

Here, we will delve into several factors. Initially, we’ll investigate the influence of disorder, subsequently examining the consequences of lattice distortion. Finally, we will elucidate the origin of exchange bias.

In a perfectly ordered double perovskite (DP), the magnetism primary governed by Ir–O–Fe(Co)–O–Ir interaction. Each transition metal ion (Fe(Co)/Ir) in an ordered DP is surrounded by six other transition metal ions $\hbox {B}^\prime$ (Ir/Fe(Co)) and exhibits AFM exchange interaction^55^. But due to the disorder this interaction chain breaks and give rise to another pair of interaction Fe–O–Fe(Co–O–Co) and Ir-O-Ir in SFI (SCI). Now discuss the new pair of interaction developed due to the disorder.

In the case of the SFI compound, if we consider the Fe–O–Fe interaction, Fe is in the $\hbox {Fe}^{3+}$ state. The electronic configuration of Fe atoms can be described as a combination of $3d^5L$ ($\hbox {Fe}^{3+}$), where the term L represents the ligand hole linked to the O 2p level. Since the Fe ions are in a high spin-state^69^, they correspond to the configurations $t_{2g}^3e_g^2$. In the case of the SCI compound, the electronic configuration for Co atoms is given by $3d^6L$ ($Co^{3+}$)^70^. The $Co^{3+}$ ions are in the ** high spin** state $t_{2g}^4 e_g^2$^71^. In both the SFI and SCI compounds, the in-plane Ir-Ir FM superexchange interaction is significantly smaller than the out-of-plane AFM interaction. For the in-plane FM interaction, electrons must hop from the Ir-$\hbox {t}_{2g}$ subband to the Ir-$\hbox {e}_g$ subband, which are separated by a substantial crystal field splitting of approximately 4 eV, making this process less probable. Conversely, electron hopping within the Ir-$\hbox {t}_{2g}$ manifold is more likely, resulting in AFM interactions along the out-of-plane direction^55^.

Among the newly introduced interactions the interaction between Fe-Fe and Co-Co pairs is highly dependent on the crystallographic direction. For Fe-Fe pairs, the out-of-plane interaction is AFM, while the in-plane interaction is FM^55,72^. However, the strength of the AFM interaction is much greater than that of the FM interaction. A similar behavior is observed for Co-Co interactions^73^. Thus, the overall interaction can be considered predominantly antiferromagnetic in nature^74^. Structural analysis indicates that the out-of-plane bond angles are significantly more distorted in the SFI compound compared to the SCI compound. This distortion weakens the AFM interactions in SFI relative to SCI. These magnetic alteration due to the ionic disorder and structural distortion are reflected in the MCE measurements, where a broad hump is observed in SFI but a sharp transition is observed in the SCI compound. Now, SFI experiences frustration due to these competing exchange interactions.

This observation supports the experimental findings, indicating a transition temperature of 60 K with a negative and larger value of $\theta _P$ (− 168K) compared to SCI’s transition temperature of 130 K with a $\theta _P$ value of − 155K.

This observation supports the experimental finding that SFI (60 K) exhibits a lower transition temperature than SCI (130 K). In the relaxation and memory experiments, we also observed that SFI exhibits glassy behavior, whereas SCI does not display such frustration.

Our experimental findings indicate the presence of EB in both compounds. The SFI compound, which exhibits a higher degree of ionic disorder and more pronounced spin-glass behavior, contains FM/AFM, FM/SG, and SG/AFM interfaces, resulting in an exchange bias of 1.86 kOe, consistent with conventional theory^75,76^. However, it is not commonly reported that a purely antiferromagnetic material can exhibit exchange bias, and the understanding of this phenomenon remains an active area of research. Recently, Asakura et al.^19^ have demonstrated for the first time that a purely antiferromagnetic system can exhibit EB, albeit the underlying mechanism is not fully understood. Surprisingly, in our SCI compound, despite the absence of magnetic memory and relaxation behavior, we observed an even higher exchange bias of 2.11 kOe. If the EB were solely attributed to disorder and spin-glass characteristics, we would expect a significantly lower value. This unexpected result suggests that, beyond spin-glass effects, an underlying antiferromagnetic mechanism also contributes to the EB in SCI. Additionally, our MCE analysis revealed strong AFM interactions in SCI. Collectively, these findings suggest that the EB in SCI predominantly originates from its AFM structure, distinguishing it as unique. Consequently, conventional theoretical frameworks are inadequate for fully explaining the EB observed in these compounds. Now, Ir is known for its strong spin-orbit coupling properties. The electronic structure calculations indicate that spin-orbit coupling enhances the noncollinear antiferromagnetic ordering, stabilizing a ground state defined by a Mott-type insulating phase in these Ir-based double perovskites^40,55^. This noncollinear antiferromagnetic structure plays a crucial role in the existence of EB in these AFM material^19^. When considering the interactions between Ir/Co and Ir/Fe, the presence of Dzyaloshinskii-Moriya (DM) interaction becomes highly plausible^62,77–80^. Dong et al. have proposed that certain noncollinear antiferromagnetic systems can exhibit EB through DM interaction. In Ir-based double perovskite systems, the DM interaction, coupled with the noncollinear magnetic structure, promotes the emergence of EB phenomena within the compound^30–32^.

### Conclusion

In summary, we utilized the solid-state reaction method to synthesize polycrystalline SFI and SCI compounds. The quality of the samples was evaluated through XRD measurements and Rietveld analysis, revealing that both compounds maintain triclinic structural symmetry. The polycrystalline nature and quality of the sample were analyzed through SEM and TEM analysis. Through HRTEM analysis, we observed the ordered arrangement of Co and Ir in SCI.

Through this structural and magnetic study of the 3d–5d element-based double perovskite, it becomes apparent that despite Fe and Co being neighboring elements in the 3d series of the periodic table, they display distinct magnetic properties influenced by their ionic disorder and lattice distortion. While both crystalline structures are triclinic, SFI exhibits more ionic disorder and structural distortion than SCI, leading to the development of FM correlation, resulting in frustration and glassy behavior in SFI.

Both compounds exhibit EB, but the origin differs significantly between them. In the case of the SFI compound, EB arises from the interactions between FM/SG, AFM/FM, and SG/AFM, resulting from the ionic disorder. However, for SCI, the origin of EB is less obvious. In MCE calculations, a sharp transition due to AFM ordering is observed in SCI. The EB characteristics can be analyzed through theoretical frameworks proposed by Dong et al., wherein the DM interaction plays a pivotal role in eliciting EB within spin-compensated AFM systems. In summary, our findings on these compounds will pave the way for future research on exchange bias in spin-compensated materials, which is crucial for potential applications in ultra-fast antiferromagnetic spintronic devices.

## Supplementary Information

Supplementary Information.

## Data Availability

The data that support the findings of this study are available from the corresponding author upon reasonable request.
