# Supplementary material for: Revealing exchange bias in spin compensated systems for spintronics applications
Source: Sci Rep. 2024 Dec 28;14:30678. doi: 10.1038/s41598-024-76130-5 (PMC11680799; doi:10.1038/s41598-024-76130-5)
Supplement: Supplementary file 1 — Supplementary Information. [file 41598_2024_76130_MOESM1_ESM.pdf]

Supplementary - Revealing Exchange Bias in Spin Compensated Systems for Spintronics Applications

Koustav Pal,[1,*] Suman Dey,[1] Aftab Alam,[2] and I.Das[1]

[1]*Saha Institute of Nuclear Physics, A CI of Homi Bhabha National Institute, Kolkata 700064, India*

[2]*Department of Physics, Indian Institute of Technology Bombay, Mumbai 400076, India*

I. LATTICE PARAMETERS OF SFI AND SCI

The lattice parameters and atomic coordinates of SFI and SCI, determined through Full Rietveld Refinement, are presented in supplementary table I.

Supplementary Table I: Crystallographic parameters of full Rietveld analysis of the compound Sr_2FeIrO_6 and Sr_2CoIrO_6 which crystallizes in triclinic I-1 space group (Group No. 2).

Composition	**Sr_2FeIrO_6**	**Sr_2CoIrO_6**
Space Group	I-1 (Group No. 2)	I-1 (Group No. 2)
$a =$	5.55(7)Å	5.54(4)Å
$b =$	5.55(3)Å	5.55(2)Å
$b =$	7.86(3)Å	7.84(1)Å
$\alpha =$	89.57(1)°	90.02(0)°
$\beta =$	90.74(3)°	89.67(1)°
$\gamma =$	89.67(1)°	90.15(5)°
Volume	242.60(5)Å^3	241.08(1)Å^3
$R_f =$	2.73	2.45
$R_{Bragg} =$	2.17	2.16
$\chi^2 =$	2.12	1.91

		**Sr_2FeIrO_6**						**Sr_2CoIrO_6**			
Atom	Wyckoff Position	x	y	z	Occupancy	Atom	Wyckoff Position	x	y	z	Occupancy
Sr	4i	0	0.52(6)	0.23(3)	1	Sr	4i	0	0.50(5)	0.25(4)	1
Fe	2a	0	0	0	0.6	Co	2a	0	0	0	0.9
Ir	2a	0	0	0	0.4	Ir	2a	0	0	0	0.1
Fe	2e	0	0	0.5	0.4	Co	2e	0	0	0.5	0.1
Ir	2e	0	0	0.5	0.6	Ir	2e	0	0	0.5	0.9
O	4i	0.96(6)	0.96(5)	0.19(8)	1	O	4i	0.97(9)	0.97(2)	0.20(2)	1
O	4i	0.23(5)	0.68(5)	0.02(0)	1	O	4i	0.27(3)	0.67(6)	0.95(7)	1
O	4i	0.83(2)	0.76(6)	0.54(2)	1	O	4i	0.83(7)	0.76(6)	0.52(5)	1

Supplementary figure 1 (a) and (b) display the Full Rietveld Refinement of SFI and SCI, respectively. In both cases, the lattice parameters, atomic coordinates, and disorder percentages were treated as free parameters to fit the XRD data. For the SFI compound, the Full Rietveld Refinement provided the best fit with 60% order, while for the SCI compound, it yielded the best fit with 90% order. We have plotted both the 60% and 90% order fittings in the XRD data for better understanding.

* koustav.pal97@gmail.com

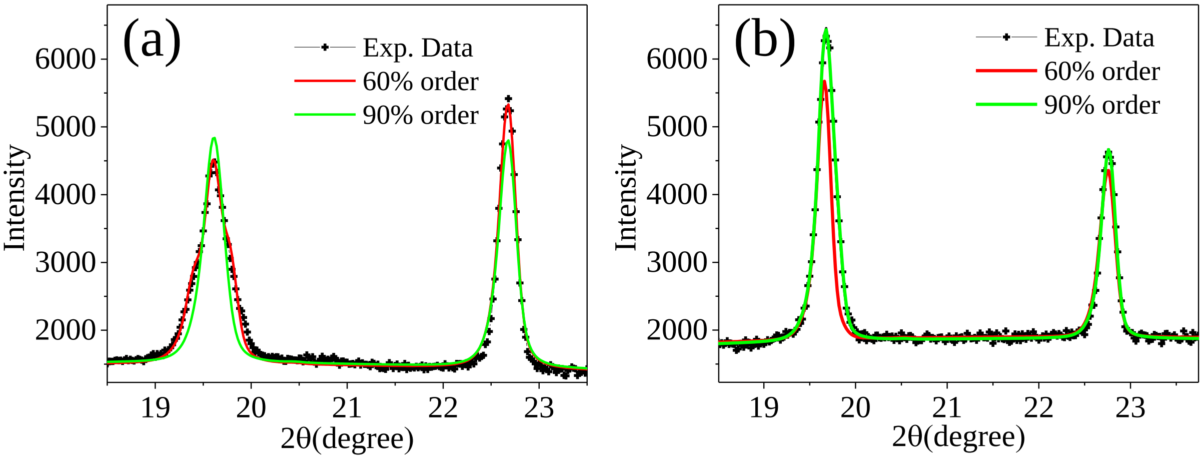

Supplementary Figure 1: Full Rietveld Refinement of (a) Sr2FeIrO6 and (b) Sr2CoIrO6 with 60% and 90% order.

II. EDAX ANALYSIS

To comprehend the chemical composition of the compounds, we conducted EDAX analysis. Supplementary figures 2(a) and (b) shows the STEM-HAADF image of the particle where the EDAX was performed is marked with orange box. Supplementary figures 2(c) and (d) display the EDAX profiles of SFI and SCI, respectively. The atomic percentages obtained from EDAX analysis for both compounds align well with their respective chemical compositions.

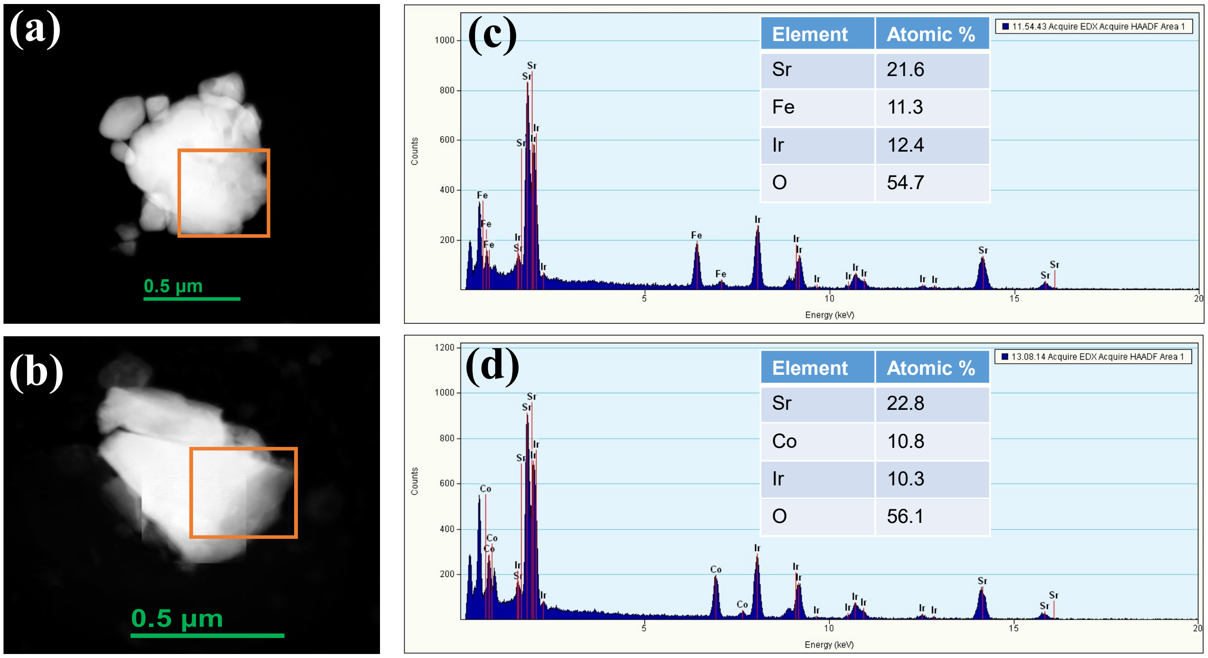

Supplementary Figure 2: STEM-HAADF image of the particle on which Elemental mapping was performed for (a) SFI, (b) SCI; EDAX profile for (c) SFI (d) SCI;

III. THEORETICAL SIMULATION

A. Model and Method

In this section, we utilized classical Monte Carlo (MC) simulations based on the isotropic Heisenberg model to explore the impact of disorder on the magnetic transition temperature and saturation magnetization. Furthermore, we investigated the interaction strength accountable for the low-temperature upturn observed in the $M(T)$ curve.

In our analysis, we opted to exclude the magnetic moments of Strontium (Sr) and Oxygen (O) due to their comparatively negligible impact compared to Ir and Fe (/Co) moments. This choice streamlined the identification of a cubic arrangement with Ir and Fe (/Co) positioned at the eight corners, thereby simplifying the computations in the MC simulation. Subsequently, we applied the classical Heisenberg model [1] as described in equation 1 within a simple cubic lattice and utilized classical MC techniques in conjunction with the Metropolis algorithm to derive the solution.

$$\mathcal{H} = J_{ab} \sum_{\langle i,j \rangle} \vec{S}_{ai}.\vec{S}_{bj} + J'_{ab} \sum_{\langle\langle i,j \rangle\rangle} \vec{S}_{ai}.\vec{S}_{bj} - h \sum_{i} S_{iz} \tag{1}$$

The terms J_{ab} and J'_{ab} denote the couplings between nearest and next-nearest neighboring spins $\vec{S}_a$ and $\vec{S}_b$, where $a, b \in$ Fe (/Co), Ir. The indices i and j represent the lattice points, and the notation $\langle\rangle$ and $\langle\langle\rangle\rangle$ signifies the summation over nearest and next-nearest neighbors, respectively. The term h denotes the external magnetic field.

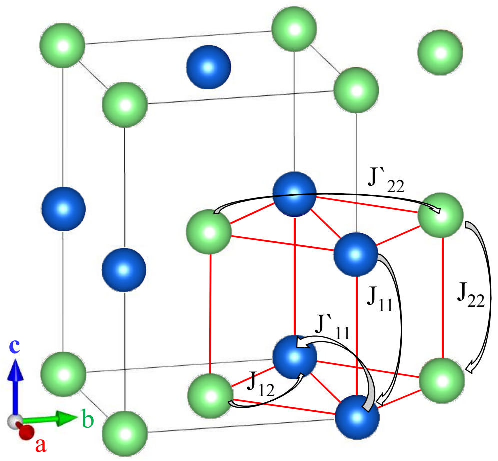

Supplementary Figure 3: Structure of with only the magnetic ions (Fe and Ir). Fe and Ir atoms are represented by blue and green spheres respectively. J_{11} (J_{22}) and J'_{11} (J'_{22}) are the couplings between nearest and next-nearest neighboring Fe (Ir) atoms. J_{12} denotes the couplings between nearest neighbor Fe and Ir atoms.

In supplementary figure 3, the illustrated interactions considered for calculation are depicted. Fe and Ir atoms are visually distinguished by blue and green colors, respectively. The strengths of the nearest neighbor (NN) interactions, Fe-Co, Fe-Ir, and Ir-Ir, are denoted by J_{11}, J_{12}, and J_{22}, respectively. Next-nearest neighbor interaction was neglected. The magnitudes of the magnetic moments of Fe and Ir atoms are indicated as $|\vec{S}_{\rm Co}| = |\vec{S}_1| = m_1$ and $|\vec{S}_{\rm Ir}| = |\vec{S}_2| = m_2$, respectively. The temperature unit is denoted by K_BT/J_{11}. Furthermore, all couplings (J_{ab}, J'_{ab}), as well as h, are expressed as ratios relative to J_{11}. In our model Hamiltonian calculations, we set the standard value for the AFM Fe-Fe interaction strength, setting $J_{11} = 1.0$, and the magnetic moment of $m_1 = 1.0$ for Fe. Based on our magnetic measurements, the magnetic moment of Ir^{5+} is determined to be approximately 50% of Fe^{3+}, leading us to use a magnetic moment of $m_2 = 0.50$ for Ir. We took J_{22} = -0.09 and J_{12} = -0.65 [2].

To address edge effects, we incorporate periodic boundary conditions. The Metropolis algorithm is employed to update spin vectors in every sweep, systematically covering each lattice site. For annealing at various temperatures, we perform 10,000 system sweeps, followed by an additional 10,000 Monte Carlo steps for computing average observables. The average magnetization, denoted as M, is measured through MC simulation and is defined as follows:

$$M = \frac{1}{N} \Big\langle \sum_i S_i \Big\rangle$$

In this investigation, a total of $N = 24^3 = 13,824$ atoms were utilized, illustrated in supplementary figure 3(b), where Fe atoms are represented in blue and Ir atoms in green. The aggregate $\sum_i S_i$ computes the collective spin across all lattice points, and the angular brackets $\langle\,\rangle$ signify an ensemble average derived from the Monte Carlo simulation.

The average magnetization was determined through two distinct methodologies. In the Zero Field Cooled (ZFC) approach, energy minimization occurred at each stage, descending from high to low temperatures without an external field. Subsequently, the system received application of the desired magnetic field value, followed by energy minimization, and the average magnetization was computed at each stage, ascending from low to high temperatures.

B. Shift in transition peak due to disorder

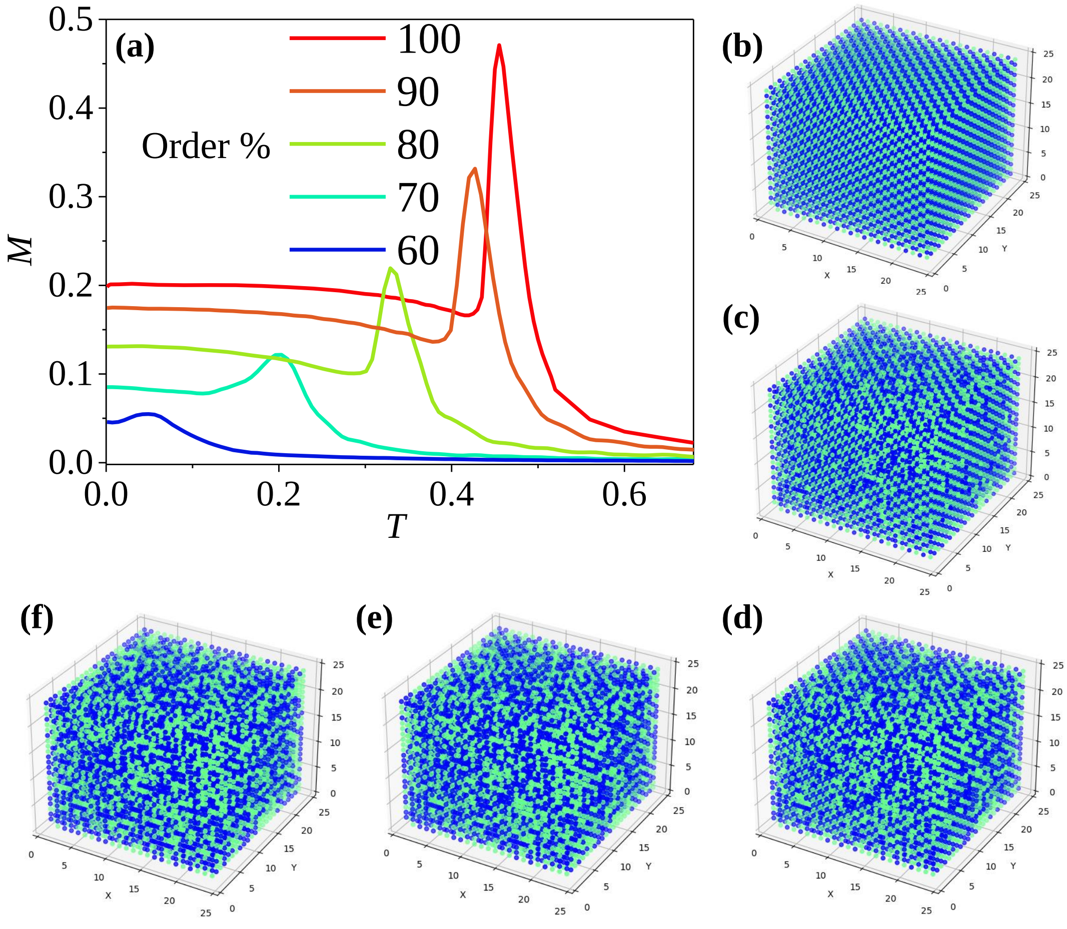

Supplementary Figure 4: (a) $M(T)$ with different percentage of order. Atomic arrangement of Fe and Ir with disorder percentage (b)100 (c) 90 (d) 80 (e) 70 (f) 60

In supplementary figure 4(a), we present the theoretically simulated ZFC curve at various disorder levels ranging from 60% to 100%. Supplementary figures 4(b) through 4(f) depict the atomic arrangement at different disorder levels. As observed in supplementary figure 4(a), both the peak magnetization value and T_N decrease with decreasing order percentage.

[1] W. Nolting and A. Ramakanth, *Quantum theory of magnetism* (Springer Science & Business Media, 2009).
[2] R. Roy and S. Kanungo, Physical Review B **106**, 125113 (2022).
